# Impact of Sulfur Fumigation on the Chemistry of Dioscoreae Rhizoma (Chinese Yam)

**DOI:** 10.1021/acsomega.3c02729

**Published:** 2023-05-30

**Authors:** Yui-Man Chan, Bo-Wen Lu, Wei-Hao Zhang, Kam-Chun Chan, Jing Fang, Han-Yan Luo, Juan Du, Zhong-Zhen Zhao, Hu-Biao Chen, Caixia Dong, Jun Xu

**Affiliations:** † School of Chinese Medicine, Hong Kong Baptist University, Hong Kong 999077, China; ‡ Tianjin Key Laboratory on Technologies Enabling Development of Clinical Therapeutics and Diagnosis, School of Pharmacy, Tianjin Medical University, Tianjin 300070, China; § Department of Pharmacognosy, College of Pharmacy, Jiamusi University, Jiamusi 154007, China; ∥ Institute of Ben Cao Gang Mu, Beijing University of Chinese Medicine, Beijing 100029, China; ⊥ Department of Metabolomics, Jiangsu Province Academy of Traditional Chinese Medicine and Jiangsu Branch of China Academy of Chinese Medical Sciences, Nanjing 210028, China

## Abstract

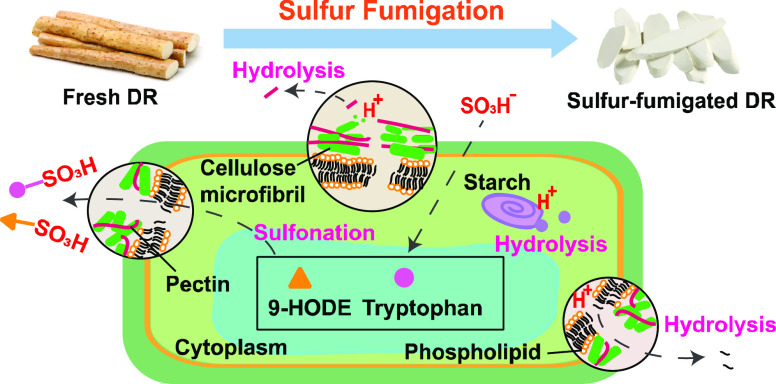

Dioscoreae Rhizoma (Chinese yam; derived from the rhizome of *Dioscorea opposita* Thunb.) (DR), commonly consumed as a food or supplement, is often sulfur-fumigated during post-harvest handling, but it remains largely unknown if and how sulfur fumigation impacts the chemistry of DR. In this study, we report the impact of sulfur fumigation on the chemical profile of DR and then the molecular and cellular mechanisms potentially involved in the chemical variations induced by sulfur fumigation. The results show that sulfur fumigation significantly and specifically changed the small metabolites (molecular weight lower than 1000 Da) and polysaccharides of DR at both qualitative and quantitative levels. Multifaceted molecular and cellular mechanisms involving chemical transformations (e.g., acidic hydrolysis, sulfonation, and esterification) and histological damage were found to be responsible for the chemical variations in sulfur-fumigated DR (S-DR). The research outcomes provide a chemical basis for further comprehensive and in-depth safety and functional evaluations of sulfur-fumigated DR.

## 1 Introduction

Dioscoreae Rhizoma (DR, Chinese yam), which is derived from the rhizome of *Dioscorea opposita* Thunb., is widely used as an ingredient in either fresh or dried form for making soup, congee, and desserts.^1^ Accumulating research has indicated that DR has multiple healthcare functions, including anti-inflammatory, antihyperlipidemia, antidiabetic, antiobesity, antidepressant, and antioxidant effects.^2^ The major types of chemical components in DR are small metabolites with a molecular weight lower than 1000 Da (e.g., amino acids and fatty acids) and polysaccharides (e.g., pectin and starch), both of which have been demonstrated to be involved in its nutritional functions.^3^

In recent decades, sulfur fumigation has been commonly used for the post-harvest processing of DR to prevent pest infestation, mold, and bacterial contamination and to give it a more attractive, whiter appearance. However, it has been found that the sulfur fumigation weakens the healthcare functions of DR, and, even worse, the consumption of sulfur-fumigated DR (S-DR) may be related to hepatotoxicity and nephrotoxicity.^4,5^ These findings indicate that sulfur fumigation changes the chemical profiles of DR qualitatively and/or quantitatively. Indeed, this indication is supported by previous studies, in which sulfur fumigation was found to affect the physicochemical properties of starch and the total contents of aromatic components in DR. For instance, the solubility of starch was found to be higher in S-DR samples than that of nonfumigated DR (NS-DR) samples, and the total flavones were more abundant in S-DR samples than those in air-dried DR samples.^6,7^ Nevertheless, the exact impacts of sulfur fumigation on the chemical profile of DR as well as the molecular and cellular mechanisms involved, both of which are necessary to thoroughly understand how sulfur fumigation impairs the nutritional value of DR, remain to be investigated.

In this study, we therefore aimed to comprehensively investigate the impact of sulfur fumigation on the chemistry of DR. First, untargeted and targeted metabolomics were developed by ultra-performance liquid chromatography coupled with quadrupole time-of-flight mass spectrometry (UPLC-QTOF-MS/MS) to characterize and compare the small metabolites in S-DR and NS-DR. Second, the physicochemical and structural properties of polysaccharides in S-DR and NS-DR were determined and compared using hyphenated chromatography, mass spectrometry, and spectroscopy. Third, chemical and histological analyses were combined to explore the molecular and cellular mechanisms involved in sulfur fumigation-induced chemical variations of DR.

## 2 Materials and Methods

### 2.1 Chemical Reagents and Materials

A PL aquagel-OH MIXED-H column (7.5 mm × 300 mm, 8 μm) was bought from Agilent (Santa Clara, CA, USA). Sulfur was purchased from Sigma-Aldrich (Steinheim, Germany). Paraffin wax was bought from Leica Biosystem (Richmond, USA). Methanol and acetonitrile were of MS-grade and purchased from Merck Co., Ltd. (Darmstadt, Germany). Formic acid (MS-grade) was provided by Sigma-Aldrich (Steinheim, Germany). Trifluoroacetic acid (TFA) (99%) was purchased from International Laboratory USA (South San Francisco, CA, USA). Ultrapure water was produced by a Milli-Q water purification system (Milford, MA, USA). Reference standards with purity ≥98%, including d-glucose (Glc), d-galactose (Gal), d-galacturonic acid (GalA), and a series of dextran standards (MW 2000, 670, 410, 270, 150, 80, 50, 12, 5, and 1 kDa), were purchased from Sigma (St. Louis, MO, USA). Other solvents and chemicals were of analytical grade.

Fresh DR samples were collected from Henan province, China. Twenty batches of commercial DR samples were purchased in Hong Kong and mainland China (Table S1). All collected samples were authenticated by Dr. Jun Xu. Voucher specimens of the DR samples were deposited at the School of Chinese Medicine, Hong Kong Baptist University.

### 2.2 Sample Preparation

#### 2.2.1 Sample Preparation of NS-DR and S-DR

S-DR samples were prepared from the fresh DR samples according to the actual practice of sulfur fumigation by farmers and wholesalers.^8^ The collected fresh DR samples were cut into slices with a thickness of about 0.3 cm. DR slices (100 g) were moistened with 10 mL of water (1:1, w/v). Sulfur powder (10 g) was heated until it burned, and then the burning sulfur and wetted DR slices were carefully put into the lower and upper layers of a desiccator, respectively. Thereafter, the desiccator was sealed for 12 h. After the sulfur fumigation, the DR slices were dried in an oven at 50 °C for 10 h to constant weight to generate the S-DR samples. The NS-DR samples were prepared by direct drying without sulfur fumigation. Both S-DR and NS-DR samples were prepared in triplicate.

#### 2.2.2 Sample Preparation for Metabolomics Analysis

Powder samples of S-DR and NS-DR (80 mesh) were accurately weighed (1 g) and ultrasonic-extracted at 45 kHz and 25 °C with 10 mL of 70% methanol for 60 min. The extracts were then centrifuged at 4000 rpm for 10 min. Supernatants were evaporated to complete dryness (NS-DR: 220 mg, S-DR: 150 mg) on a rotary evaporator at 55 °C. Residues were redissolved with 3 mL of 70% methanol. Thereafter, the extracts were centrifuged again at 4000 rpm for 10 min, and the supernatants obtained were then filtered through a 0.45 μm PTFE syringe filter before UPLC-QTOF-MS/MS analysis.

#### 2.2.3 Sample Preparation for Polysaccharides Analysis

DR powders (80 mesh) were accurately weighed (100 g) and extracted by reflux with 14-fold boiling water (100 °C) (1.4 L × 1.5 h × 2 times). The extracts were centrifuged at 5500 rpm for 15 min, and the supernatants were collected and combined. The extracted solutions were then precipitated by adding ethanol to make a final concentration of 80% and kept at 4 °C for 24 h. After centrifugation at 5500 rpm for 15 min, the precipitate was washed with ethanol three times and dried in a water bath at 60 °C to remove residual ethanol. The generated precipitate was washed with Sevag reagent (isoamyl alcohol and CHCl_3_ in 1:4 ratio). After that, the precipitate was dissolved in water and then dialyzed with molecular cut-off 3.5 kDa for 48 h. The dialyzate was lyophilized, yielding the crude polysaccharides.

### 2.3 Metabolomics Analysis

#### 2.3.1 Liquid Chromatography

Chromatographic separation was performed on an Agilent 1290 UPLC system (Agilent Technologies, Santa Clara, CA, USA) equipped with a binary pump, thermostatic column compartment, autosampler, and degasser. An aliquot of sample (3 μL) was injected into an ACQUITY UPLC BEH C18 column (2.1 mm × 100 mm, 1.7 μm; Waters, Milford, MA, USA) operated at 40 °C. The separation was achieved using a linear gradient elution with 0.1% formic acid in water (A) and 0.1% formic acid in acetonitrile (B) at a flow rate of 0.4 mL/min. The UPLC elution condition was optimized as follows: 15–30% B (0–2 min), 30–34% B (2–5 min), 34–36% B (5–12 min), 36–60% B (12–16 min), 60% B (16–24 min), 60–100% B (24–26 min), 100% B (26–29 min), 100–15% B (29–29.1 min), and isocratic at 15% (29.1–32 min).

#### 2.3.2 Mass Spectrometry

MS data were collected using an Agilent 6540 QTOF mass spectrometer (Agilent Technologies) equipped with a JetStream electrospray ion (ESI) source. Data acquisition software was MassHunter Qualitative Analysis B.06.00 (Agilent Technologies). The optimized operating parameters in negative ion mode were as follows: nebulizing gas (N_2_) flow rate at 7 L/min, nebulizing gas temperature at 300 °C, JetStream gas flow at 7 L/min, sheath gas temperature at 350 °C, nebulizer pressure at 40 psi, capillary voltage at 3000 V, skimmer at 65 V, Octopole RFV at 600 V, and fragmentor voltage at 130 V. An MS/MS technique was applied to provide parallel alternating scans for acquisition at low collision energy to obtain precursor ion information or at a ramping of high collision energy to acquire a full-scan accurate mass data of fragments and precursor ions and to obtain neutral loss information. The collision energies for auto MS/MS analysis were 20 and 35 V.

#### 2.3.3 Method Validation

A quality control (QC) sample was created by combining 100 μL of 70% methanol extracts from each sample. The QC sample was performed six times per day to condition the instrument before the main analytical run began. The DR, blanks, and QC samples were examined in three analytical blocks during the main analytical run, each required about 24 h of the instrument time. Each block had seven segments, each of which contained nine samples that were randomly chosen and enclosed by a QC sample and blank sample. The QC samples that were injected between DR samples were employed in the processing of metabolomics analysis data as for further quality assurance.

#### 2.3.4 Multivariate Statistical Analysis

Raw data files of UPLC-QTOF-MS/MS in negative ion mode were converted to comma-separated value files (CSV) and compound exchange files (CEF) in a DA reprocessor (Agilent Technologies), for the purpose of peak findings, filtering, and alignment. Followings were the parameters of DA method: retention time range, 0–32 min; mass range, 100–1000 Da; absolute height greater or equal to 5000 counts; [M-H]^−^ as allowed ion species; isotope peak spacing tolerance at *m*/*z* 0.0025 plus 7.0 ppm; quality score greater or equal to 80. The obtained CSV files were trimmed down to three columns (mass-to-charge ratio, retention time, intensities) and processed by MetaboAnalyst 5.0 (Sainte-Anne-de-Bellevue, Québec, Canada). Mass Profiler Professional B0.2 software (MPP, Agilent Technologies) was used for analyzing CEF files.

For data preprocessing, mass tolerance was set to 0.25 *m*/*z* and retention time tolerance was set to 30.0 s after uploading the CSV files to MetaboAnalyst 5.0. For data processing, an automatically generated peak intensity table was produced with each feature labeled with the mass-to-charge ratio and retention time. The table also consisted of missing value processing, data filtering, and data normalization. Features with an excessive number of missing values were deleted from missing value processing to improve downstream analysis. The option of “Removing features with > 50% missing values” was selected. The option of “Replace by a small value” was chosen to estimate the remaining missing values. When modeling the data for metabolomics, data filtering is crucial for identification and removal of unsuitable variables. To adjust the differences between samples, “Normalization by sum” was chosen in the data normalization process.

After processing, SIMCA 14.1 (Umetrics, Ume, Sweden) was used to visualize the normalized data. The chemical profiles of the NS-DR and S-DR samples were compared using unsupervised principal component analysis (PCA) and partial least-squares-discriminant analysis (PLS-DA). Pareto scaling was used to build PCA models, whereas PLS-DA models utilized both log transformation and Pareto scaling. Cross-validations were employed by using the default software options to verify the effectiveness and reliability of the models. Examination of the parameters R^2^X (cum.) and R^2^Y (cum.), for PCA model and PLS-DA model, respectively, and Q^2^ (cum.) was conducted, in which R^2^X (cum.) and R^2^Y (cum.) indicated goodness of fit, whereas Q^2^ (cum.) revealed the accuracy of the prediction. To determine whether the PLS-DA models were overfitted, permutation tests were employed. When the variable importance in projection (VIP) value of a metabolite was more than 1, it was considered to be largely contributed to the chemical difference between NS-DR and S-DR samples.

After uploading the CEF files to MPP, qualitative analysis of MS features was achieved by using “MS Experiment Creation Wizard”, an automated data analysis module. To remove redundant data, all the data were first normalized at 75% percentile. To ensure 50% of compounds were present in at least one studied group, frequency analysis was set to 50%. Then, the unpaired *t* test was used to filter compounds that were significantly different between NS-DR and S-DR samples. The *p* value cut-off of 0.05 was then used in Benjamini–Hochberg multiples testing correction. To identify the metabolites that were different between NS-DR and S-DR samples, fold change cut-off was set to 2 for listed compounds. Data was re-examined by recursion analysis to confirm the presence of each entity in the samples. Extracted ion chromatograms (EIC) were re-extracted to perform the recursion analysis. To remove the false positive and false negative, peaks of the resulted EIC were examined.^9^

### 2.4 Polysaccharide Analysis

#### 2.4.1 Determination of Total Sugar, Protein, and Uronic Acid Contents

Phenol-sulfuric acid method was performed to determine the content of total sugar by using d-galactose as the standard.^10^ Proteins were examined by recording the absorption of samples (50 μg/mL) at 200–400 nm on a Hitachi U-2900 UV/VIS spectrophotometer (Hitachi High-Technologies, Japan). Uronic acid content was determined by an *m*-hydroxyldiphenyl assay using d-galacturonic acid as the standard.^11^

#### 2.4.2 Determination of Molecular Weight

The molecular weight of DR polysaccharides was identified and determined by an HPGPC method on an Agilent 1200 HPLC (Santa Clara, CA, USA) system equipped with a PL aquagel-OH MIXED-H column (7.5 mm × 300 mm, 8 μm) and a refractive index detector (RID, Agilent) as described in our previous study.^12^ NaNO_3_ (0.1 M) was chosen as the mobile phase with a flow rate of 0.6 mL/min, and the column temperature was maintained at 35 °C. The standard dextrans with different molecular weights (Mw) (2000, 670, 410, 270, 150, 80, 50, 12, 5, and 1 kDa) were used for the construction of the molecular weight-retention time calibration curve.

#### 2.4.3 FT-IR Spectroscopy Analysis

DR polysaccharides (2 mg) were ground with dried KBr powder (100 mg) and then pressed into tablets. The FT-IR spectra of DR polysaccharides were scanned using Nicolet 380 FT–IR spectrophotometer (Nicolet, Thermo Scientific, USA) with a scan range from 4000 to 400 cm^–1^.

#### 2.4.4 Monosaccharide Composition Analysis

DR polysaccharides (1 mg) were dissolved in 0.3 mL of water and then hydrolyzed by 0.3 mL of 2 M TFA at 120 °C for 2 h under nitrogen pressure. The hydrolyzed samples were dried to remove the excess TFA under nitrogen gas and then converted into alditol acetates for GC–MS analysis.^12^ GC–MS analysis was achieved by an Agilent GC–MS 7890A-5975 instrument with a fused silica capillary column (HP-5 MS, 30 m × 0.25 mm, 0.25 μm, Agilent, USA) and helium as the carrier gas. The temperature of injection and detector was maintained at 280 °C. The oven temperature was set to rise from 160 to 190 °C at 2 °C/min, then to 240 °C at 5 °C/min, and kept at 240 °C for 5 min.

#### 2.4.5 Methylation Analysis

Glycosidic linkages of NS-DR and S-DR samples were analyzed according to previous study.^13^ Partly methylated alditol acetates were produced by hydrolyzing the products with 2 M TFA at 120 °C for 2 h, followed by reduction with NaBD_4_ and acetylation with acetic anhydride. GC–MS analysis was conducted on an Agilent GC–MS instrument using an HP-5 MS-fused silica capillary column (30 m × 0.25 mm, 0.25 μm, Agilent). During injection, the column temperature was set at 120 °C, increased to 280 °C at 4 °C/min, and then kept at 280 °C for 5 min. Helium was used as the carrier gas. Identification of the compounds that corresponded to each peak was performed by examining the obtained mass spectra. The molar ratio of each residue was determined based on peak areas.

### 2.5 Residual Sulfur Dioxide (SO_2_) Determination

Residual SO_2_ in DR samples was quantified according to the method described in the Chinese Pharmacopeia (2020 edition).^14^ Each DR powder sample was accurately weighed (10.0 g) and placed into a round bottom flask (1 L). After that, 400 mL of water and 10 mL of 6 M hydrochloric acid were added into the sample. Nitrogen gas was supplied into the flask at a flow rate of 0.2 L/min. The flask was heated gently in a heating mantle for 1.5 h. The hydrogen peroxide solution (3%, v/v) absorbed SO_2_ that produced during boiling, which was then measured by sodium hydroxide titration until the yellow color did not change within 20 s. The content of SO_2_ was calculated according to the volume of 0.01 M NaOH used during the titration after boiling (1 mL of sodium hydroxide titration solution corresponds to 0.032 mg of SO_2_).

### 2.6 Histological Analysis

Permanent slides of the NS-DR and S-DR samples were prepared for histological analysis. Fresh NS-DR and S-DR samples were fixed in formalin–acetic–alcohol (FAA) for 24 h. The samples were then dehydrated using a series of ethanols (50, 60, 70, 80, 90, and 100%) and passed through a graded series of xylene-ethanol solutions up to 100% xylene. After that, samples were transferred to molten paraffin, and specimen blocks were prepared using molds. Samples embedded in the specimen blocks were sectioned at a thickness of 15 μm using Leica RM2255 fully automatic rotary microtome (Leica Microsystems, Wetzlar, Germany). The sections were then stained by safranin and counterstained with fast green.^15^ The stained sections were observed by a Leica DM5000 B light microscope (Leica Microsystems, Wetzlar, Germany). Parenchyma cells were measured by imageJ software version 1.53k (National Institutes of Health, Bethesda, MD, United States). For parenchyma cell count, 5 views were randomly selected for each group, parenchyma cells with complete cell walls were marked in the digital images. The damage was expressed as a cell wall broken rate, which was quantified as described previously^16^ with modification:
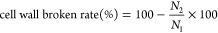
where *N*_1_ is the average number of cells from the NS-DR sample and *N*_2_ is the average number of unbroken cells from the S-DR sample.

### 2.7 Quantitative Statistical Analysis

All the quantitative data were presented as mean ± standard deviation (SD) of triplicate determinations. Prism 8.0 (GraphPad Software, San Diego, CA, USA) was used for the construction of graphs related to the quantification results of metabolomics, residual SO_2_ determination, and histological analysis. The two-tailed Student’s *t*-test was used for statistical analysis. A significant difference was indicated when *p* < 0.05, 0.01, or 0.001. Heatmap of correlation was achieved by Prism 8.0.

## 3 Results and Discussion

### 3.1 Untargeted Metabolomics Analysis

#### 3.1.1 Chemical Profiling of NS-DR and S-DR

**Figure 1:**
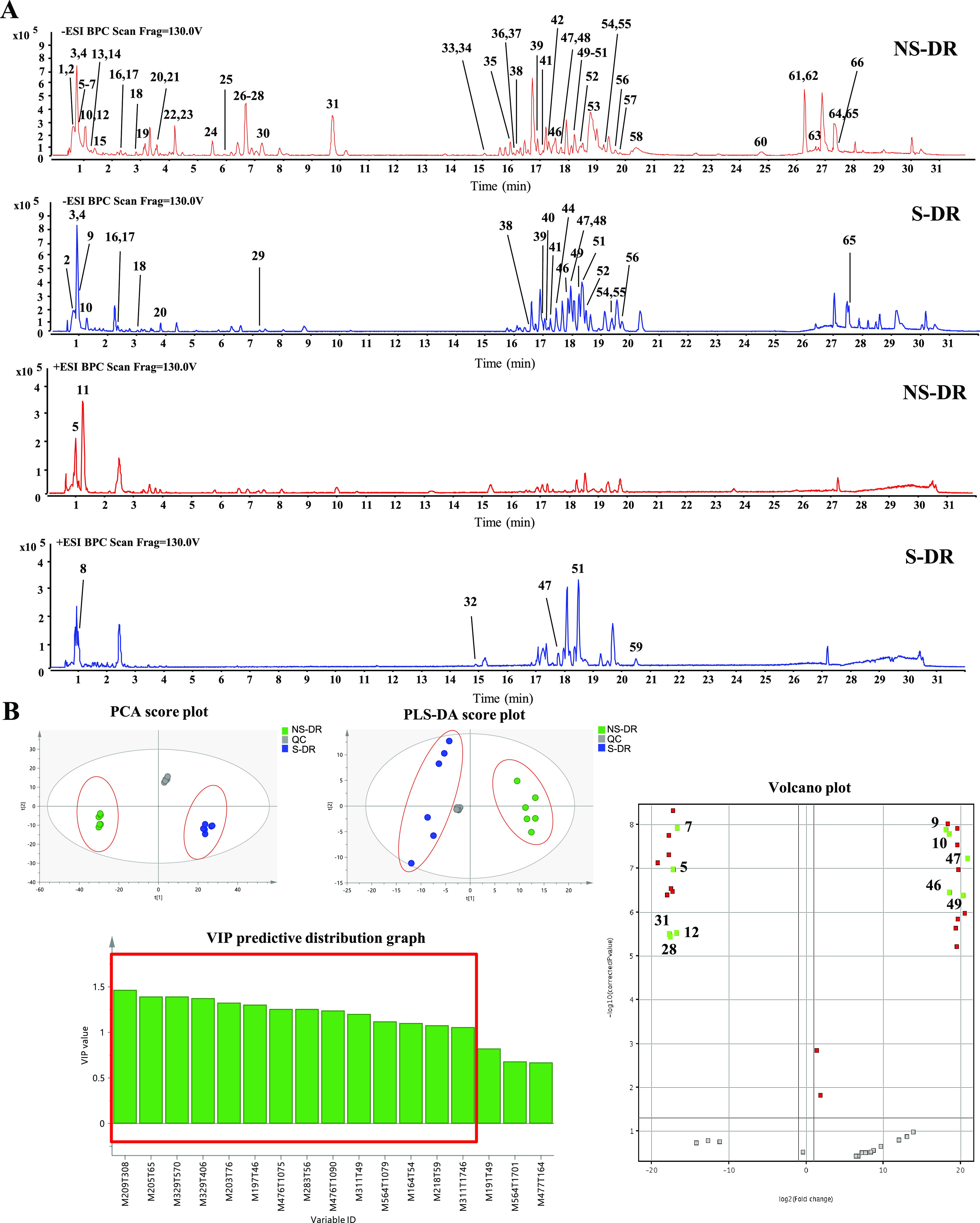
(A) Representative BPI chromatograms of NS-DR and S-DR samples in negative and positive ion modes. The peak numbers are the same as those in Table S2. (B) Multivariate statistical analysis of NS-DR and S-DR samples. Significant components with VIP value greater than 1 are highlighted with red square in the VIP predictive distribution graph. Significant components are marked with its peak number in the volcano plot (*n* = 3).

Chemical profiling of the NS-DR and S-DR samples was conducted in both negative and positive ion modes of UPLC-QTOF-MS/MS. As shown in Table S2, a total of 66 compounds, including fatty acids, amino acids, organic acids, lysophosphatidylethanolamines (LysoPE), lysophosphatidylcholines (LysoPC), and phenols, were tentatively identified from DR samples through matching the empirical formula and fragment ions with those of the published known compounds.^3,17−26^ Base peak intensity (BPI) chromatograms (Figure 1A) show that the chemical profiles of S-DR and NS-DR are significantly different. To be specific, compared to those in NS-DR, the intensities of many peaks (i.e., peaks 2, 3, 5–7, 11–15, 19, 21–28, 30–38, 42, 43, 45, 50, 53, 54, 57–65) in S-DR were substantially reduced or even disappeared, whereas some others (i.e., peaks 8, 9, 10, 16, 18, 20, 29, 39–41, 44, 46, 47, 49, 51, 55, 56) were increased or newly detected. The result clearly demonstrates that sulfur fumigation quantitatively and qualitatively changed the profile of small metabolites in DR.

#### 3.1.2 Multivariate Statistical Analysis

The UPLC-QTOF-MS/MS data were processed using multivariate statistical analysis, including PCA and PLS-DA, to further study the effect of sulfur fumigation on the small metabolites in DR. Data obtained in negative ion mode were used for the multivariate statistical analysis, as more peaks were detected in negative ion mode than that of positive ion mode. MetaboAnalyst was used to process 1552 peaks from 18 data sets of samples (6 NS-DR, 6 S-DR, and 6 QC samples). After data filtering, 407 features were discovered and then imported into SIMCA 14.1 to perform PCA and PLS-DA analysis. To illustrate the degree of clustering or dispersion across various sample groups by lowering the dimensionality of the data sets, the results of PCA and PLS-DA were shown as score plots.^27^ According to the PCA and PLS-DA score plots shown in Figure 1B, all the samples fell well into the 95% tolerance region of confidence level. For the PCA model, R^2^X (cum.) and Q^2^ (cum.) with four components were 0.964 and 0.709, respectively (Table S3), which exhibits good fit and decent predictive ability based on the criterion of R^2^X (cum.) near to 1, Q^2^ (cum.) larger than 0.5, and their difference within 0.2.^28^ PLS-DA was employed to further investigate the difference between NS-DR and S-DR samples. For PLS-DA model, R^2^Y (cum.) and Q^2^ (cum.) with four components were 0.962 and 0.899, respectively (Table S3), which demonstrated that the obtained PLS-DA model possessed good modeling quality. To further validate the model, permutation tests (*n* = 200) were achieved. As shown in Figure S1, the intercepts of R^2^ and Q^2^ were smaller than 1, i.e., R^2^ = (0.0, 0.376), Q^2^ = (0.0, −0.295), which indicated that the PLS-DA model was repeatable. Both the PCA and PLS-DA score plots (Figure 1B) showed that NS-DR and S-DR samples were clearly clustered into two groups, which further demonstrated that sulfur fumigation changed the chemical profile of small metabolites in DR.

To explore the potential chemical markers that can discriminate between NS-DR and S-DR samples, the VIP predictive distribution graph and volcano plot were obtained from SIMCA 14.1 and MPP, respectively. As shown in Figure 1B, 14 components with a VIP value greater than 1 were identified as important variables to differentiate between NS-DR and S-DR samples (marked in red square in the VIP predictive distribution graph). The volcano plot (Figure 1B) showed that 27 ions were found after statistical analysis with a filter frequency of 50%, univariate significant analysis at a *p* value ≤0.05, and fold change cut-off ≥2.0 between NS-DR and S-DR samples. Finally, 10 out of 27 ions (i.e., peaks 5, 7, 9, 10, 12, 28, 31, 46, 47, 49, Figure 1B) were selected as the potential chemical markers with VIP value greater than 1.^29^

#### 3.1.3 Structural Elucidation of the Potential Chemical Markers

The 10 chemical markers were identified by matching the empirical molecular formula and fragment ions with those of published known compounds. Structural elucidation of the chemical markers was demonstrated as follows with selected examples.

**Figure 2:**
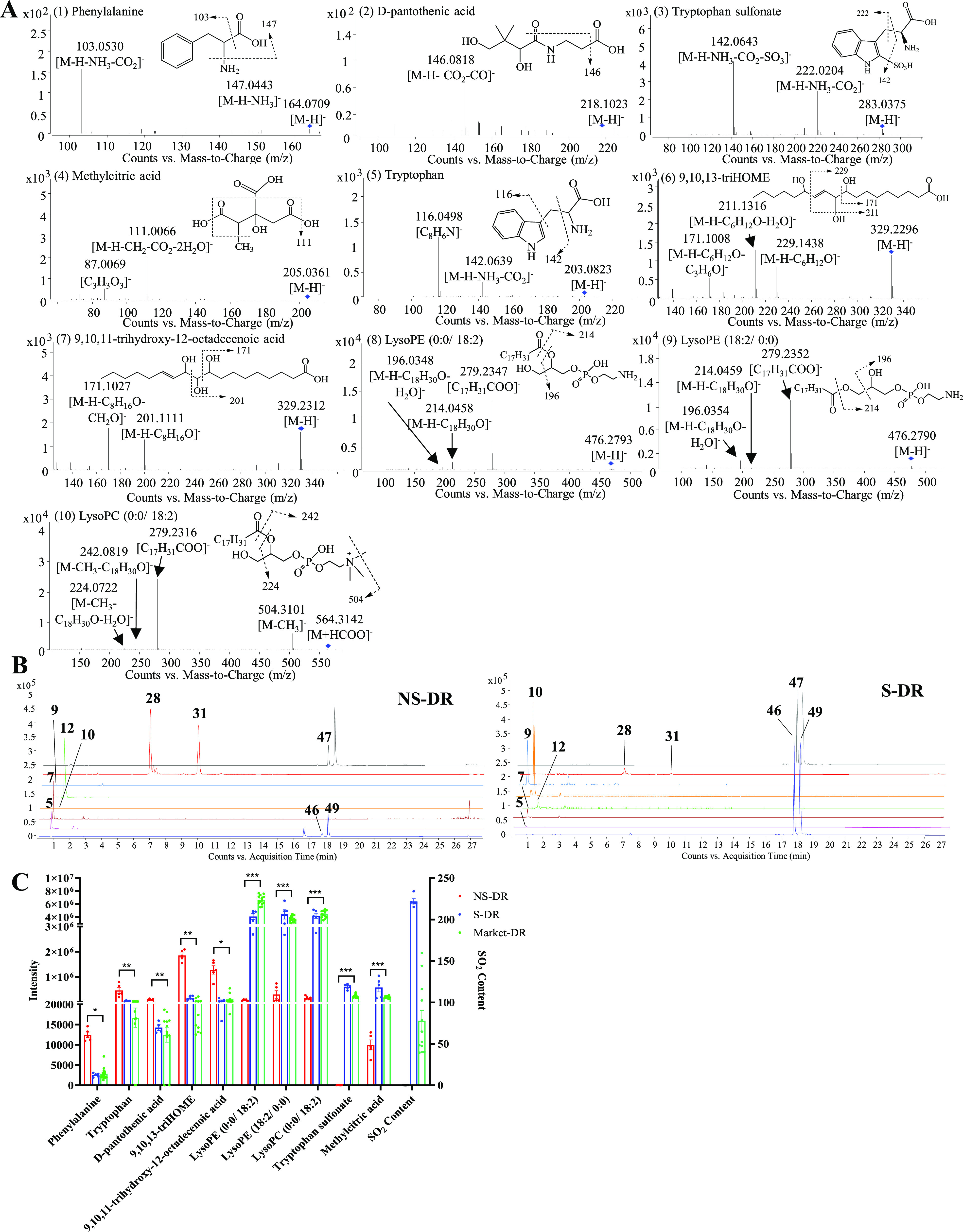
(A) Mass spectra and (B) EICs of the 10 chemical markers, and (C) contents of the 10 chemical markers and SO_2_ in NS-DR, S-DR, and Market-DR samples. 5: phenylalanine, 7: d-pantothenic acid, 9: tryptophan sulfonate, 10: methylcitric acid, 12: tryptophan, 28: 9,10,13-triHOME, 31: 9,10,11-trihydroxy-12-octadecenoic acid, 46: LysoPE (0:0/18:2), 47: LysoPC (0:0/18:2), 49: LysoPE (18:2/0:0). Peak intensities are expressed as mean ± SD. *p < 0.05, **p < 0.01, and ***p < 0.001 (*n* = 3).

Peak 12 showed [M-H]^−^ at *m*/*z* 203.0823 with the molecular formula C_11_H_12_N_2_O_2_ in negative ion mode, which could be further dissociated to form fragment ions at *m*/*z* 142.0639 [M-H-NH_3_-CO_2_]^−^ and *m*/*z* 116.0498 [C_8_H_6_N]^−^.^19^ Therefore, peak 12 was tentatively identified as tryptophan. Peak 9 showed [M-H]^−^ at *m*/*z* 283.0375 (C_11_H_12_N_2_O_5_S), 80 Da more than that of tryptophan (*m*/*z* 203.0823). As shown in Figure 2A, fragment ions at *m*/*z* 222.0204 [M-H-NH_3_-CO_2_]^−^ and *m*/*z* 142.0643 [M-H-NH_3_-CO_2_-SO_3_]^−^ were observed. Therefore, peak 9 was tentatively assigned as tryptophan sulfonate.^19^ Both peaks 28 and 31 yielded a precursor ion [M-H]^−^ at *m*/*z* 329.23 with the molecular formula C_18_H_34_O_5_ and further fragmented into *m*/*z* 171.10, which suggested that a hydroxyl group is present at position C9.^21^ Peak 28 also produced fragment ions at *m*/*z* 229.1438 and *m*/*z* 211.1316, which correspond to the loss of C_6_H_12_O and water, respectively. Therefore, peak 28 was tentatively identified as 9,10,13-trihydroxy-11-octadecenoic acid (9,10,13-triHOME). Peak 31 generated fragment ions at *m*/*z* 201.1111 [M-H-C_8_H_16_O]^−^ and *m*/*z* 171.1027 [M-H-C_8_H_16_O-CH_2_O]^−^. Therefore, peak 31 was tentatively identified as 9,10,11-trihydroxy-12-octadecenoic acid.^21^ Peaks 46 and 49 were regio-isomers with identical molecular formula C_23_H_44_NO_7_P (*m*/*z* 476.28). As shown in Figure 2A, both spectra displayed fragment ions at *m*/*z* 279.23, *m*/*z* 214.04, and *m*/*z* 196.03, which correspond to a carboxylate 18:2 anion and neutral loss of an acyl chain as ketene (*m*/*z* 214) or fatty acid (*m*/*z* 196).^22^ Therefore, peaks 46 and 49 were tentatively assigned as regio-isomers of LysoPE (18:2). The major difference between peaks 46 and 49 was the intensity of the two fragment ions, *m*/*z* 214 and *m*/*z* 196. The relative signal intensity ratio 196/214 of the sn_1_ isomer should be larger than 1.^22^ Thus, peak 49 with the relative signal intensity ratio larger than 1 was proposed to be the sn_1_ isomer, while peak 46 was suggested to be the sn_2_ isomer. Based on the above interpretation, peaks 46 and 49 were tentatively identified as LysoPE (0:0/18:2) and LysoPE (18:2/0:0), respectively. With the same strategy, peak 47 was tentatively identified to be LysoPC (0:0/18:2).

### 3.2 Targeted Metabolomics Analysis

The EICs of the 10 chemical markers were obtained to display and compare the differences of the chemical markers between NS-DR and S-DR samples (Figure 2B). The peak areas were used for semiquantification of the 10 chemical markers to investigate the content variations caused by sulfur fumigation of DR. As shown in Figure 2C, the contents of five chemical markers (phenylalanine, tryptophan, d-pantothenic acid, 9,10,13-triHOME, and 9,10,11-trihydroxy-12-octadecenoic acid) were reduced whereas four chemical markers (LysoPE (0:0/18:2), LysoPE (18:2/0:0), LysoPC (0:0/18:2), and methylcitric acid) were increased after sulfur fumigation. In addition, one chemical marker, namely, tryptophan sulfonate, was newly detected in S-DR samples.

The contents of the 10 chemical markers were then examined in the 20 batches of commercial DR samples. The results showed that the contents of the 10 chemical markers in the commercial DR samples were similar with those in the S-DR samples (*p* > 0.05) but were significantly different from those in the NS-DR samples (*p* < 0.05). To be specific, five chemical markers (phenylalanine, tryptophan, d-pantothenic acid, 9,10,13-triHOME, and 9,10,11-trihydroxy-12-octadecenoic acid) in the commercial samples were significantly lower (*p* < 0.01 or *p* < 0.05) than those in the NS-DR samples, whereas the contents of the four chemical markers (LysoPE (0:0/18:2), LysoPE (18:2/0:0), LysoPC (0:0/18:2), and methylcitric acid) were significantly higher (*p* < 0.001) than those of the NS-DR samples (Figure 2C). In addition, tryptophan sulfonate was detected in all of the commercial samples (Figure 2C). These findings strongly suggest that the commercial samples were sulfur-fumigated. To confirm this, SO_2_ determination assay was adopted. The results showed that SO_2_ was detected, ranging from 4.15 ± 1.80 to 159.67 ± 9.66 mg/kg, in all of the commercial samples (Figure 2C), which evidenced that the commercial samples were sulfur-fumigated.

Figure S2 shows the heatmap of correlation between the contents of chemical markers and SO_2_. The results indicate that the five chemical markers (phenylalanine, tryptophan, d-pantothenic acid, 9,10,13-triHOME, and 9,10,11-trihydroxy-12-octadecenoic acid) decreased by sulfur fumigation were negatively correlated with the SO_2_ content, with the coefficient (*r*) ranging from −0.47 to −0.09, while the other five markers including LysoPE (0:0/18:2), LysoPE (18:2/0:0), LysoPC (0:0/18:2), tryptophan sulfonate, and methylcitric acid were positively correlated with the SO_2_ content (*r* between 0.43 and 0.71). The correlations further confirmed the content variations of the 10 chemical markers associated with sulfur fumigation.

### 3.3 Polysaccharide Analysis

**Figure 3:**
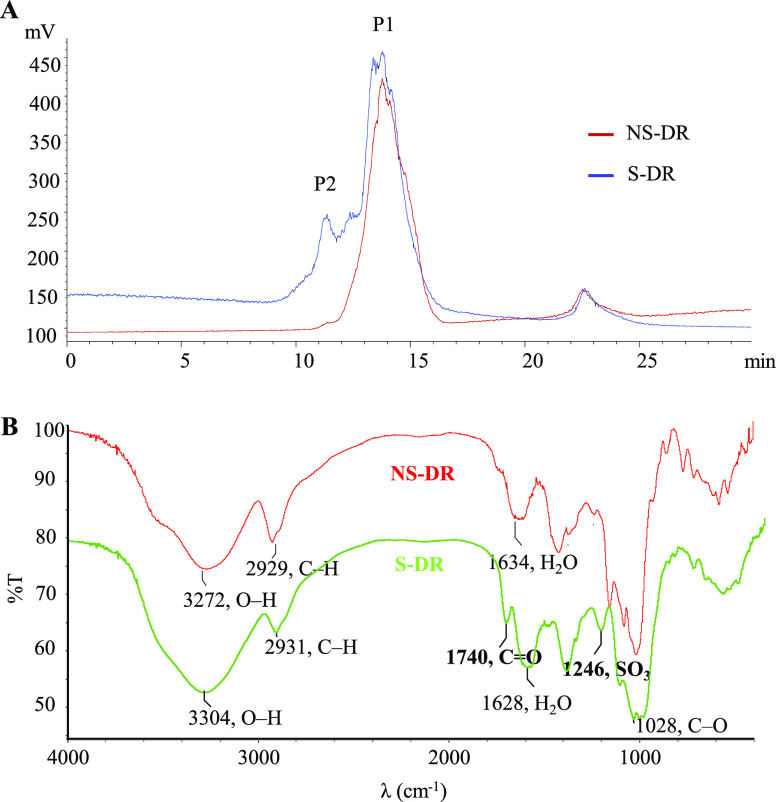
(A) HPGPC chromatograms and (B) FT-IR spectra of NS-DR and S-DR polysaccharides.

The extraction yield of polysaccharides in NS-DR was 29.15%, and it contained 84.8% total sugars and 9.9% uronic acids (Table 1). NS-DR polysaccharides exhibited a single symmetrical peak (P1) on the HPGPC chromatogram (Figure 3A), with a molecular weight of 9.2 kDa. A weak UV absorption peak at 260–280 nm was detected, which suggested that NS-DR polysaccharides contained a small amount of protein. The monosaccharide composition analysis shows that NS-DR polysaccharides were composed of three kinds of monosaccharides, namely, Glc, Gal, and GalA, with the molar ratio of 85.6:10.3:4.1 (Table 1). Methylation analysis indicated that NS-DR polysaccharides contained a large amount of l,4-Glc*p* (71.4%) and a few other types of linkages, including terminal Glc*p* (*t*-Glc*p*) (7.9%), 1,4,6-Glc*p* (2.7%), 1,4-Gal*p* (11.5%), and 1,4-Gal*p*A (4.7%) (Table 1). The structural characteristics of polysaccharides in DR were consistent with previous reports as follows and led to the conclusion that NS-DR polysaccharides comprise a large amount of glucans (e.g., galactoglucan and mannoglucan) and a few pectins and galactans.^30^ A water-soluble polysaccharide mainly composed of 1,4-Glc*p* with about 6% 1,6-Gal*p* was isolated from DR.^31^ Another study isolated a mannoglucan from DR polysaccharides, which mainly consisted of 1,4-Glc*p* with a molar ratio of 72.0%.^32^ DR polysaccharides also included galactans comprised of 96.7% 1,4-Gal*p* and a few *t*-Gal*p*.^33^ A pectin isolated from DR polysaccharides contained 59.1% homogalacturonan and 38.1% rhamnogalacturonan I regions, and both regions were mainly consisted of 1,4-Gal*p*A.^34^

**Table 1:** Chemical Composition, Monosaccharide Composition, and Linkage Types of NS-DR and S-DR Polysaccharides^a^

	samples	
item	NS-DR	S-DR
***chemical composition* (*%*, *w*/*w*)**		
total sugar	84.8 ± 1.7	68.1 ± 11.4^**^
uronic acid	9.9 ± 0.4	17.1 ± 0.7*
***monosaccharide composition* (*%*, *mol*)**		
Glc	85.6 ± 0.8	31.0 ± 1.5^***^
Gal	10.3 ± 1.1	34.4 ± 0.9^***^
GalA	4.1 ± 0.4	34.6 ± 1.5^***^
***linkage type* (*%*, *mol*)**		
*t*-Glc*p*	7.9 ± 0.3	4.8 ± 0.3^**^
1,4-Glc*p*	71.4 ± 1.4	22.8 ± 1.3^***^
1,4,6-Glc*p*	2.7 ± 0.1	3.0 ± 1.0
		
1,4-Gal*p*	11.5 ± 1.1	34.3 ± 0.1^***^
1,3,4-Gal*p*	1.8 ± 0.5	0.6 ± 0.2
1,3,6-Gal*p*	-	1.0 ± 0.1
		
1,4-Gal*p*A	4.7 ± 0.1	33.5 ± 0.2^***^

a -: undetectable; Glc = glucose; Gal = galactose; GalA = galacturonic acid; *p < 0.05, **p < 0.01, and ***p < 0.001 as compared to NS-DR. Data are expressed as mean ± SD (*n* = 3).

The physicochemical and structural properties of polysaccharides in S-DR were characterized using the same methods. Compared to NS-DR, S-DR had a lower extraction yield (19.42%). The uronic acid content in S-DR (17.1%) was higher (*p* < 0.05) than NS-DR (9.9%), while the total sugar content in S-DR (68.1%) was lower (*p* < 0.01) than NS-DR (84.8%) (Table 1). A peak (P2) was newly detected at 11 min (the molecular weight of about 573.6 kDa) on the HPGPC chromatogram of S-DR polysaccharides (Figure 3A). In the FT-IR spectrum (Figure 3B), the strong and wide absorption peaks at around 3300 cm^–1^ could be attributed to the O–H stretching vibration of the sugar ring and the absorption at around 2900 cm^–1^ was resulted from the stretching vibration of the C–H bond. The absorption peaks at around 1630 cm^–1^ were due to the water binding in samples, and the absorption peaks at 1030 cm^–1^ were derived from the bending vibrational modes of C–O stretching in pyranose. Most importantly, the spectrum of S-DR showed a newly detected weak absorption peak at 1246 cm^–1^, which was attributed to the stretching vibration of −SO_3_ group. The absorption in the S-DR spectrum at 1740 cm^–1^ attributed to the carbonyl group was obviously stronger in comparison with NS-DR, further evidencing the higher content of uronic acid in S-DR. Monosaccharide composition analysis showed that the molar ratio of Gal greatly increased from 10.3% in NS-DR to 34.4% in S-DR (*p* < 0.001), and that of GalA increased from 4.1% in NS-DR to 34.6% in S-DR (*p* < 0.001). In contrast, the molar ratio of Glc significantly reduced from 85.6% in NS-DR to 31.0% in S-DR (*p* < 0.001) (Table 1). Methylation analysis demonstrated that sulfur fumigation increased the amount of 1,4-Gal*p*A from 4.7% in NS-DR to 33.5% in S-DR (*p* < 0.001), while reduced the amount of 1,4-Glc*p* from 71.4% in NS-DR to 22.8% in S-DR (*p* < 0.001) (Table 1). These results clearly demonstrate that the polysaccharides extracted from S-DR are quantitatively and qualitatively different from those from NS-DR.

### 3.4 Molecular and Cellular Mechanisms Potentially Involved in the Chemical Variations in Sulfur-Fumigated DR

We have revealed that sulfur fumigation significantly impacts the chemical components of DR especially small metabolites and polysaccharides. Next, the molecular and cellular mechanisms potentially involved in the chemical variations were further explored. Sulfur fumigation created an acidic environment in the presence of water and SO_2_.^35^ Glycosidic bonds and ester bonds in certain components of DR are easily hydrolyzed in an acidic environment. For instance, acid hydrolysis of ester bonds in phospholipids could result in the increase of lysophospholipids (e.g., LysoPE (18:2/0:0)) in S-DR samples.^36^ Glycosidic linkages in glucans are easily hydrolyzed to be oligomers or monomers, thereby reducing the content of Glc and 1,4-Glc*p*.^37^ In addition, the amount of 1,4-Gal*p*A was increased by sulfur fumigation. Since 1,4-Gal*p*A was detected in pectins but not in glucans and galactans,^32−34^ it is therefore suggested that sulfur fumigation may promote the extraction of pectins. The mechanism may be related to the mild acid hydrolysis of ester bonds and/or glycosidic bonds in pectins releasing polygalacturonic acids.^38^ In addition to acidic hydrolysis, sulfur fumigation can also trigger sulfation or sulfonation of original compounds to produce sulfur-containing derivatives.^39^ For example, tryptophan may transform into tryptophan sulfonate; if so, this would explain the reduction of tryptophan and the new generation of tryptophan sulfonate.^40^ The chemical transformation of 9-hydroxyoctadecadienoic acid (9-HODE) into 9-HODE sulfite may contribute to the decrease of 9-HODE and the formation of 9-HODE sulfite. Moreover, a weak absorption peak that represents the stretching vibration of −SO_3_ group was detected in the FT-IR spectrum of S-DR polysaccharides. This implies that a small amount of sulfated polysaccharides are produced during sulfur fumigation.^41^ Other chemical mechanisms can also contribute to the chemical variations of DR.^42^ For example, esterification of carboxylic hydroxyl groups may be associated with the reduction of phenylalanine, 9,10,13-triHOME, and 9,10,11-trihydroxy-12-octadecenoic acid.^43^

**Figure 4:**
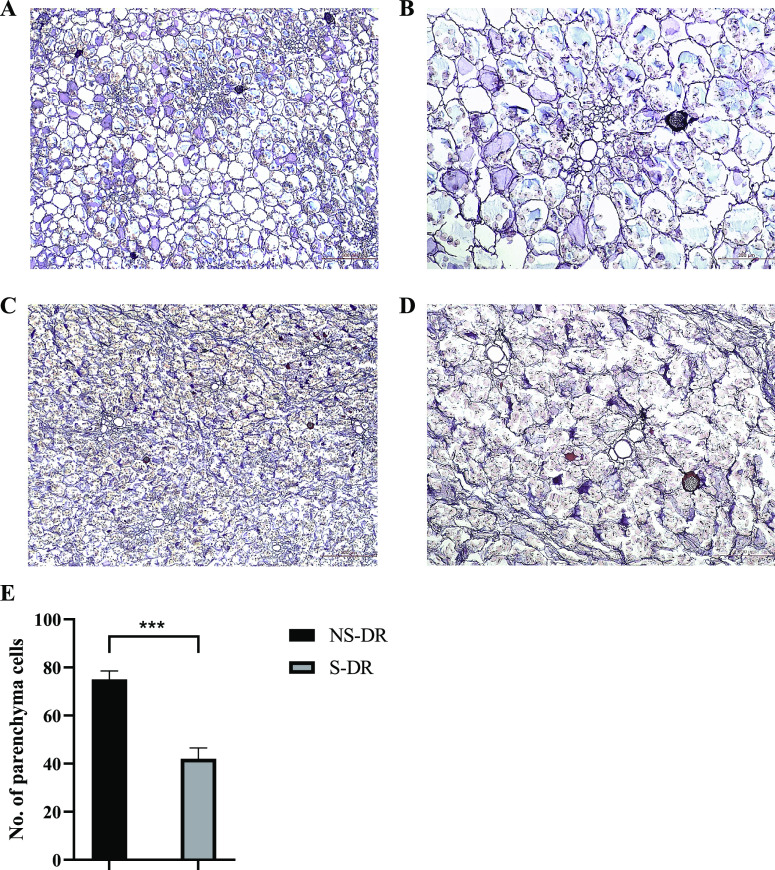
Photomicrographs of histological cross sections of DR (15 μm thick): (A) NS-DR (40×), (B) NS-DR (100×), (C) S-DR (40×), (D) S-DR (100×), and (E) cell count on parenchyma cells. Parenchyma cells with the complete cell wall are selected from digital images (100×) from 5 views for each group using ImageJ software. Results are expressed as mean ± SD. ***p < 0.001. (*n* = 3).

**Figure 5:**
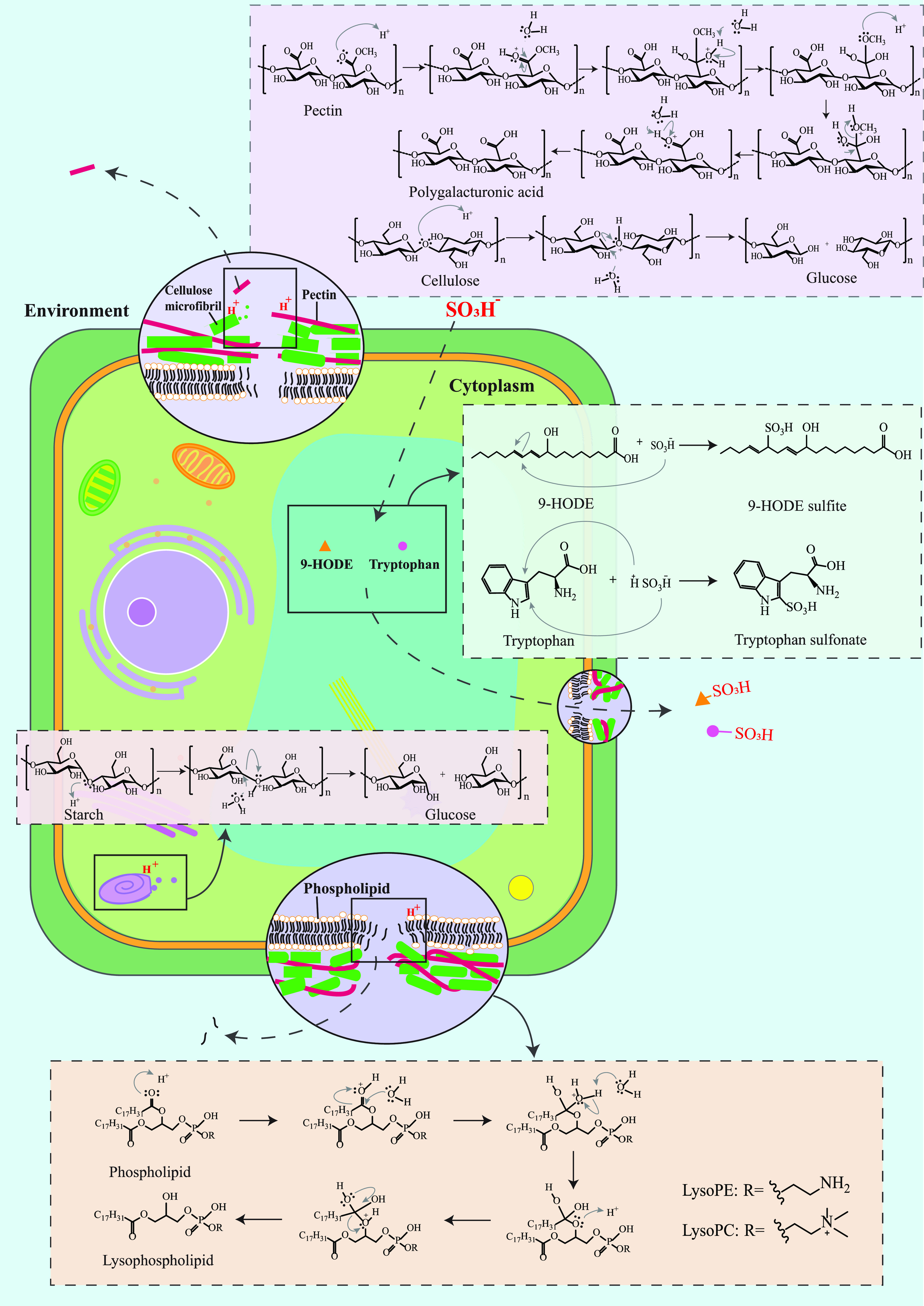
Molecular and cellular mechanisms potentially involved in the chemical variations of DR by sulfur fumigation.

It was further noticed that some of the compounds in DR affected by sulfur fumigation were the components of cellular structures. For example, lysophospholipids are the components of cell membranes, while pectins are the components of the cell wall.^44,45^ We therefore speculated that the chemical variations caused by sulfur fumigation were related to changes of the histological structure in DR. Thus, the cellular histomorphology of NS-DR and S-DR were compared. As shown in Figure 4A,B, the central ground tissue of NS-DR was mainly composed of parenchyma cells with starch granules stored inside; this pattern is consistent with previous study.^15^ Parenchyma cells with rigid cell walls were observed in NS-DR, whereas distorted parenchyma cells with broken cell walls were observed in S-DR (Figure 4C,D). NS-DR (75 ± 3.51) had a larger number of complete parenchyma cells than S-DR (42 ± 4.55) (p < 0.001), and the cell wall breakage rate of S-DR was 44% (Figure 4E). These results demonstrated that cell walls were damaged by sulfur fumigation, which may be due to the acid hydrolysis of cell wall components (e.g., glucans and pectins).^46^ Structural damage to cells may in turn facilitate the release of components stored inside the cells including both small metabolites and polysaccharides, and this release would further contribute to the chemical variations caused by sulfur fumigation. For instance, the detection of P2, a peak with molecular weight higher than P1, in the HPGPC chromatogram of S-DR polysaccharides may be related to the release of macromolecules from the broken cell walls.^47^ In summary, multifaceted molecular and cellular mechanisms including chemical reactions (e.g., acidic hydrolysis, sulfonation, and esterification) and histological damage could be triggered by sulfur fumigation, and these processes collectively resulted in the chemical variations in sulfur-fumigated DR (Figure 5).

As aforementioned, polysaccharides, amino acids, and fatty acids are the major nutritional components of DR.^48^ Thus, any quantitative and/or qualitative changes of these components by sulfur fumigation may affect the safety and healthcare functions of DR. In such a case, further investigation is warranted. For example, tryptophan is an essential amino acid required for protein synthesis and it is a precursor of key biomolecules (serotonin, melatonin, tryptamine, etc.) vital for human health (e.g., regulation of immune response, mood and, antioxidant defense).^49^ In addition, tryptophan can be related to the hydroxyl radical-scavenging activity of DR.^50^ Reduction of tryptophan caused by sulfur fumigation may affect the synthesis of key biomolecules and scavenging power on free radicals, thus various nutritional functions of DR (e.g., anti-inflammatory, antioxidant, and antidepressant effect) can be influenced.^5^ Furthermore, the toxicity and bioactivity of tryptophan sulfonate, the sulfur-containing derivative of tryptophan in S-DR, are still unknown. Another example is polysaccharides. Relationships between the structure and function of natural pectins or celluloses have been previously reported;^51^ if and how the variations in chemical properties of S-DR’s pectins and celluloses affect their functions are questions yet to be answered.

## 4 Conclusions

In this study, the impact of sulfur fumigation on the chemistry of DR was investigated, and then the mechanisms involved in the sulfur fumigation-induced chemical variations were explored. The results showed that sulfur fumigation significantly changed the chemical profiles of DR, particularly the small metabolites and polysaccharides. Many of these molecules are responsible for DR’s functional attributes as a food supplement. The molecular mechanisms involved include acidic hydrolysis, sulfonation, and esterification. Histological damage was also found to potentially contribute to the chemical variations of DR caused by sulfur fumigation. This study provides a chemical basis for further comprehensive and in-depth safety and function evaluations of S-DR.

## Data Availability

Data are included in the article or in the Supporting Information.

## Supporting Information Available

(Figure S1) Result of permutation test (*n* = 200) for checking if the PLS-DA model of NS-DR and S-DR were overfitted; (Figure S2) heatmap of correlation between the content of chemical markers and SO_2_; metabolites with negative correlation were represented in red, and metabolites with positive correlation were represented in blue; 9,10,13-triHOME = 9,10,13-trihydroxy-11-octadecenoic acid, LysoPE = lysophosphatidylethanolamine, LysoPC = lysophosphatidylcholine; ^#^*p* < 0.05, ^##^*p* < 0.01, and ^###^*p* < 0.001; (Table S1) information of the 20 batches of commercial DR samples; (Table S2) identification of the major components in DR samples by UPLC-QTOF-MS/MS; and (Table S3) summary for PCA and PLS-DA models (PDF)
