# Supplementary material for: Impact of Sulfur Fumigation on the Chemistry of Dioscoreae Rhizoma (Chinese Yam)
Source: ACS Omega. 2023 May 30;8(23):21293–304. doi: 10.1021/acsomega.3c02729 (PMC10269262; doi:10.1021/acsomega.3c02729)
Supplement: Supplementary file 1 — ao3c02729_si_001.pdf [file ao3c02729_si_001.pdf]

Supplementary Material

Impact of sulfur fumigation on the chemistry of Dioscoreae Rhizoma (Chinese yam)

Yui-Man Chan [a, #], Bo-Wen Lu [b, c, #], Wei-Hao Zhang [a], Kam-Chun Chan [a], Jing Fang [a], Han-Yan Luo [a], Juan Du [c], Zhong-Zhen Zhao [d], Hu-Biao Chen [a*], Caixia Dong [b*], Jun Xu [a, e*]

[a] *School of Chinese Medicine, Hong Kong Baptist University, Hong Kong*

[b] *Tianjin Key Laboratory on Technologies Enabling Development of Clinical Therapeutics and Diagnosis, School of Pharmacy, Tianjin Medical University, Tianjin 300070, China*

[c] *Department of Pharmacognosy, College of Pharmacy, Jiamusi University, Jiamusi 154007, China*

[d] *Institute of Ben Cao Gang Mu, Beijing University of Chinese Medicine, Beijing 100029, China*

[e] *Department of Metabolomics, Jiangsu Province Academy of Traditional Chinese Medicine and Jiangsu Branch of China Academy of Chinese Medical Sciences, Nanjing 210028, China*

*Corresponding author:
Jun Xu, Ph D

School of Chinese Medicine, Hong Kong Baptist University, Hong Kong

Tel: 852-34112423, E-mail: davidxujun@hkbu.edu.hk (J. Xu)

Cai-Xia Dong, Ph D

Tianjin Key Laboratory on Technologies Enabling Development of Clinical Therapeutics and Diagnosis, School of Pharmacy, Tianjin Medical University, Tianjin 300070, China

Tel: 86-183-2247-3708, Email: dongcaixia@tmu.edu.cn (C.-X. Dong)

Hu-Biao Chen, Ph D

School of Chinese Medicine, Hong Kong Baptist University, Hong Kong

Tel: 852-34112060, Email: hbchen@hkbu.edu.hk (H.-B. Chen)

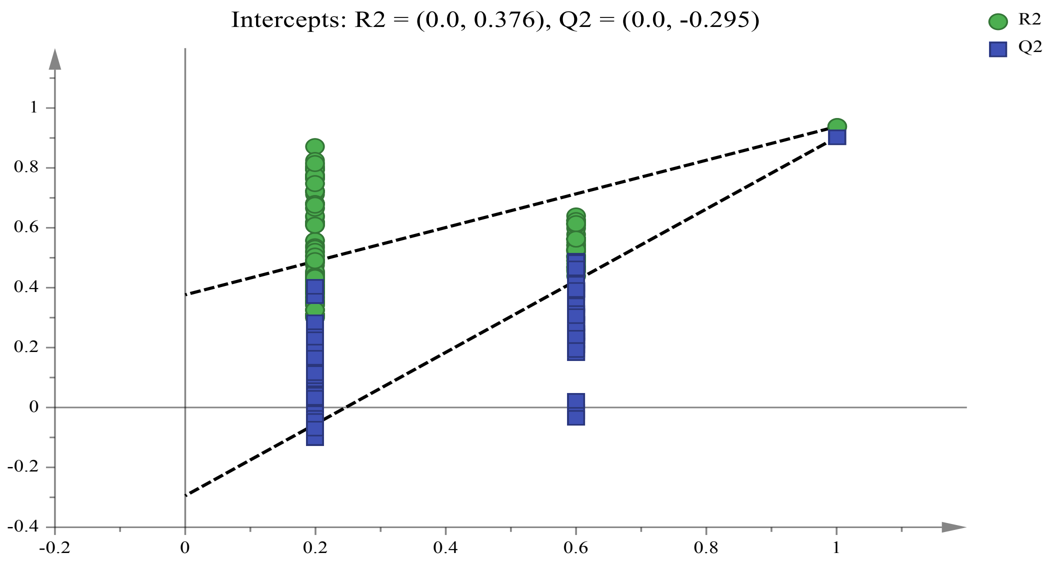

Figure S1 Result of permutation test (*n* = 200) for checking if the PLS-DA model of NS-DR and S-DR were overfitted.

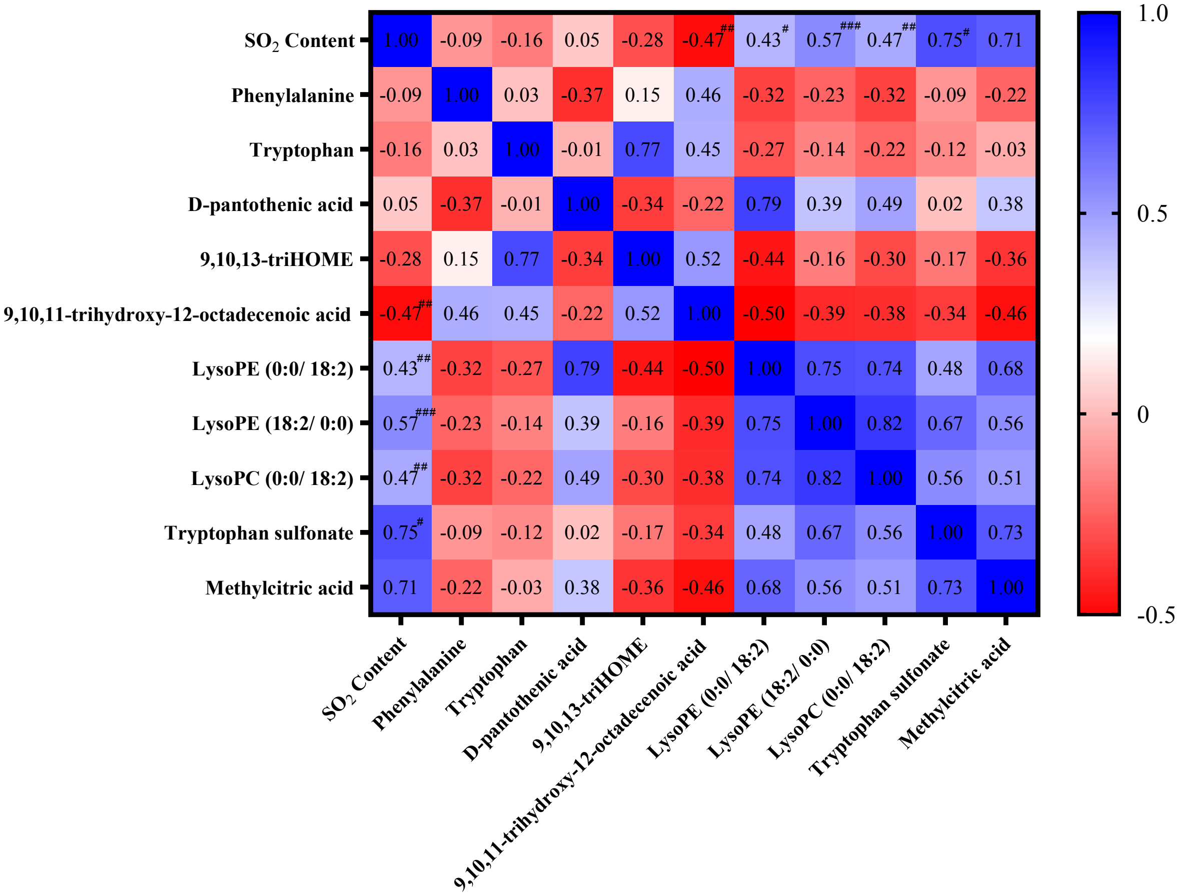

Figure S2 Heatmap of correlation between the content of chemical markers and SO_2. Metabolites with negative correlation were represented in red, and metabolites with positive correlation were represented in blue. 9,10,13-triHOME = 9,10,13-trihydroxy-11-octadecenoic acid; LysoPE = Lysophosphatidylethanolamine; LysoPC = Lysophosphatidylcholine. $^{\#}p < 0.05$, $^{\#\#}p < 0.01$, and $^{\#\#\#}p < 0.001$.

Table S1 Information of the 20 batches of commercial DR samples

No.	Collect date	Collection location
1	07-11-2020	Hefei, Anhui, China
2	07-11-2020	Chengdu, Sichuan, China
3	09-11-2020	Kunming, Yunnan, China
4	10-11-2020	Dalian, Liaoning, China
5	10-11-2020	Wuhan, Hubei, China
6	10-11-2020	Xiamen, Fujian, China
7	10-11-2020	Zibo, Shandong, China
8	11-11-2020	Loudi, Hunan, China
9	11-11-2020	Hangzhou, Zhejiang, China
10	13-11-2020	Guangzhou, Guangdong, China
11	18-02-2021	Kowloon City, Hong Kong
12	31-05-2021	Kai Tak, Hong Kong
13	31-05-2021	Kai Tak, Hong Kong
14	31-05-2021	San Po Kong, Hong Kong
15	05-07-2021	Cheung Chau, Hong Kong
16	02-08-2021	Sheung Wan, Hong Kong
17	30-08-2021	Central, Hong Kong
18	18-09-2021	Tsing Yi, Hong Kong
19	18-09-2021	Tsing Yi, Hong Kong
20	10-01-2022	Shan Shui Po, Hong Kong

Table S2 Identification of the major components in DR samples by UPLC-QTOF-MS/MS

No.	RT (min)	Identification	Molecular Formula	Ion species	Ions (*m/z*)	Mass (*m/z*)	Error (ppm)	MS/MS fragments (*m/z*) (% abundance)	Chemical classification	Fold Change
1	0.60	Arginine	$C_6H_{14}N_4O_2$	$[M-H]^-$	173.104	174.111	-3.76	156.2533 (4.7), 111.0081 (100)	Amino acid	1.08
2	0.70	Dehydroascorbic acid	$C_6H_6O_6$	$[M-H]^-$	173.009	174.016	-2.60	112.8775 (4.57), 111.0081 (100)	Vitamin	0.45
3	0.74	Isocitric acid	$C_6H_8O_7$	$[M-H]^-$	191.020	192.027	-0.50	191.0549 (0.32), 129.0190 (2.78), 111.0081 (100)	Organic acid	0.72
4	0.86	Citric acid	$C_6H_8O_7$	$[M-H]^-$	191.020	192.027	0.84	191.0011 (0.16), 129.0202 (2.61), 111.0800 (100)	Organic acid	1.06
5	0.90	Phenylalanine	$C_9H_{11}NO_2$	$[M-H]^-$	164.071	165.079	-3.06	164.0709 (6.54), 147.0443 (42.64), 103.0530 (100)	Amino acid	0.45
				$[M+H]^+$	166.087	165.080	5.93	120.0819 (66.27), 103.0566 (100)	Amino acid	0.63
6	0.90	5-Acetamidopentanoic acid	$C_7H_{13}NO_3$	$[H+CH_3COO]^-$	218.103	159.089	-2.85	116.0747 (8.94), 98.9563 (6.7), 59.0152 (100)	Fatty acid	0.72
7	0.90	D-Pantothenic acid	$C_9H_{17}NO_5$	$[M-H]^-$	218.103	219.110	-2.17	146.0818 (100)	Vitamin	0.41
8	0.96	N-Acetyl-L-Tryptophan	$C_{13}H_{14}N_2O_3$	$[M+K]^+$	285.062	246.098	-8.01	188.0766 (40.87), 159.098 (10.86), 132.084 (7.25)	Amino acid	1.71
9	0.96	Tryptophan sulfonate	$C_{11}H_{12}N_2O_5S$	$[M-H]^-$	283.041	284.048	-4.28	283.0375 (7.56), 222.0204 (59.74), 142.0643 (100)	Amino acid derivatives	17.81
10	1.05	Methylcitric acid	$C_7H_{10}O_7$	$[M-H]^-$	205.036	206.043	0.54	111.0066 (100), 87.0069 (18.94)	Vitamin derivatives	16.39
11	1.16	Tryptophanium	$C_{11}H_{13}N_2O_2$	$[M+H]^+$	206.105	205.097	-0.39	188.0766 (6.86), 143.0754 (38.25), 118.0667 (78), 117.0592 (61.1), 115.056 (100)	Amino acid	0.02
12	1.17	Tryptophan	$C_{11}H_{12}N_2O_2$	$[M-H]^-$	203.082	204.089	-2.25	203.0823 (1.54), 142.0639 (23.99), 116.0498 (100)	Amino acid	0.08
13	1.35	Ferulic acid	$C_{10}H_{10}O_4$	$[M+HCOO]^-$	239.055	194.057	-4.30	149.0611 (90), 134.3025 (4.08)	Phenolic acid	0.88
14	1.38	Caffeic acid	$C_9H_8O_4$	$[M+CH_3COO]^-$	239.055	180.041	-4.70	239.0558 (10.51), 179.0348 (83.61), 135.0443 (100)	Phenolic acid	0.54
15	1.49	2-Isopropylmalic acid	$C_7H_{12}O_5$	$[M-H]^-$	175.061	176.068	-1.21	115.0391 (100), 113.0594 (42.45)	Organic acid	0.50
16	2.27	α-Hydroxycinnamic acid	$C_9H_8O_3$	$[M-H]^-$	163.040	164.047	-1.93	162.6959 (0.67), 120.0533 (23.43), 119.0492 (100)	Phenolic acid	1.58
17	2.32	N-Acetyl-L-phenylalanine	$C_{11}H_{13}NO_3$	$[M-H]^-$	206.082	207.089	-3.39	164.0650 (100), 147.0434 (41.79)	Amino acid	1.09
18	3.09	Azelaic acid	$C_9H_{16}O_4$	$[M-H]^-$	187.097	188.105	-1.23	187.8345 (15.09), 125.0955 (100), 123.0771 (25.13)	Organic acid	1.41

19	3.21	Cis-N-coumaroyltyramine	$C_{17}H_{17}NO_3$	$[M-H]^-$	282.112	283.119	-5.31	282.1091 (89.35), 163.3136 (10.94)	Organic acid	0.36
20	3.63	Tans-Cinnamic acid	$C_9H_8O_2$	$[M+CH_3COO]^-$	207.067	148.052	-1.42	207.0729 (100), 102.9408 (44.64)	Organic acid	1.47
21	3.67	Hannokinol	$C_{19}H_{24}O_4$	$[M-H]^-$	315.159	316.166	-5.70	315.1588 (100), 107.0472 (4.97)	Phenols	0.30
22	4.30	1,7-Bis(4-hydroxyphenyl)-1,5-epoxy- 3-hydroxyheptane	$C_{19}H_{22}O_4$	$[M-H]^-$	313.144	314.151	-3.68	163.0393 (0.21), 151.0639 (0.87), 149.0598 (100), 147.0429 (0.69), 145.6610 (0.24), 107.0490 (2.88)	Diarylheptanoid	0.07
23	4.38	Demethylbatatasin IV	$C_{14}H_{14}O_3$	$[M-H]^-$	229.086	230.094	-3.56	185.9502 (3.38), 183.9914 (9.81), 159.0807 (6.58), 123.0446 (100)	Stilbenoid	0.82
24	5.61	9,12,13-Trihydroxy-10,15-octadecadienoic acid	$C_{18}H_{32}O_5$	$[M-H]^-$	327.216	328.224	-4.12	327.2169 (70.67), 291.196 (15.2), 229.1446 (72.67), 211.1337 (100), 183.1402 (14.94), 127.1119 (9.8)	Fatty acid	0.06
25	6.08	(3R,5R)-1,7-bis(4-hydroxy-3-methoxyphenyl)-3,5-heptanediol	$C_{21}H_{28}O_6$	$[M-H]^-$	375.181	376.188	-1.82	375.1805 (34.92), 193.0680 (9.3), 191.0741 (100)	Phenols	0.21
26	6.74	Nigakilactone I	$C_{21}H_{28}O_6$	$[M-H]^-$	375.182	376.189	0.14	375.1805 (34.92), 201.1094 (28.08), 135.0065 (2.6), 191.0741 (100)	Phenols	0.89
27	6.74	Hannokinol	$C_{19}H_{24}O_4$	$[M+CH_3COO]^-$	375.182	316.168	0.20	191.0734 (10.19), 119.9784 (3.32), 107.9546 (100)	Phenols	0.41
28	6.79	9,10,13-trihydroxy-11-octadecenoic acid (9,10,13-triHOME)	$C_{18}H_{34}O_5$	$[M-H]^-$	329.232	330.240	-3.40	329.2296 (100), 229.1438 (56.27), 211.1316 (93.34), 171.1008 (41.62)	Fatty acid	0.35
29	7.19	9-hydroxyoctadecadienoic acid sulfite (9-HODE sulfite)	$C_{18}H_{32}O_6S$	$[M-H]^-$	375.185	376.192	0.99	375.1849 (46.87), 295.2278 (81.03), 277.2162 (48.83), 191.0747 (100), 183.1394 (66.87)	Fatty acid derivatives	13.21
30	7.21	9,12,13-Trihydroxy-10-octadecenoic acid	$C_{18}H_{34}O_5$	$[M-H]^-$	329.232	330.239	-5.53	329.2313 (60.38), 229.1420 (17.1), 171.1026 (100)	Fatty acid	0.05
31	9.82	9,10,11-trihydroxy-12-octadecenoic acid	$C_{18}H_{34}O_5$	$[M-H]^-$	329.233	330.240	-2.78	329.2312 (82.97), 311.2212 (10.87), 201.1111 (72.02), 171.1027 (100), 127.1106 (25.93)	Fatty acid	0.01
32	14.76	L-Prolyl-L-Phenylalanine	$C_{14}H_{18}N_2O_3$	$[M+Na]^+$	285.118	262.129	-8.95	139.0574 (100), 153.0719 (36.92), 115.0553 (21.19)	Amino acid	0.23

33	15.09	13-Hydroperoxy-6,9,11-octadecatrienoic acid	$C_{18}H_{30}O_4$	$[M-H]^-$	309.207	310.214	-2.00	309.2075 (100), 291.1908 (3.57), 174.9562 (46.62)	Fatty acid	0.27
34	15.15	10-Hydroperoxy-8,12-octadecadienoic acid	$C_{18}H_{32}O_4$	$[M-H]^-$	311.222	312.229	-1.97	311.2168 (31.7), 293.2106 (97.66), 153.5759 (5.58), 139.1103 (19.82)	Fatty acid	0.22
35	15.89	11-hydroperoxyl-9,12-octadecadienoic acid	$C_{18}H_{32}O_4$	$[M-H]^-$	311.222	312.229	-2.79	311.2203 (50.93), 295.2178 (2.18), 293.2086 (100)	Fatty acid	0.83
36	16.13	(9Z)-12,13-Dihydroxyoctadec-9-enoic acid	$C_{18}H_{34}O_4$	$[M-H]^-$	313.237	314.245	-3.45	313.2373 (100), 295.2214 (8.15), 183.1402 (5.68), 127.1129 (4.68)	Fatty acid	0.40
37	16.13	10-Heptadecenoic Acid	$C_{17}H_{32}O_2$	$[M+HCOO]^-$	313.237	268.239	-3.73	313.2373 (100), 267.2232 (1.38)	Fatty acid	0.91
38	16.29	13-Hydroperoxy-9,11-octadecadienoic acid	$C_{18}H_{32}O_4$	$[M-H]^-$	311.222	312.229	-3.80	311.2212 (24.55), 295.2232 (8.36), 277.2076 (38.19), 171.1018 (100)	Fatty acid	0.26
39	16.91	LysoPC 18:3/ LysoPC (0:0/ 18:3)	$C_{26}H_{48}NO_7P$	$[M+HCOO]^-$	562.316	517.318	1.79	562.3149 (0.77), 278.2224 (24.06), 277.2189 (100), 242.0415 (0.21), 224.0728 (2.6)	Lysophosphatidylcholine	17.48
40	17.13	LysoPE 18:3	$C_{23}H_{42}NO_7P$	$[M-H]^-$	474.263	475.271	1.55	474.2656 (14.33), 277.2192 (100)	Lysophosphatidylethanolamine	8.22
41	17.21	LysoPC 18:3(2n isomer)/ LysoPC (18:3/ 0:0)	$C_{26}H_{48}NO_7P$	$[M+HCOO]^-$	562.316	517.317	1.07	562.3102 (0.59), 502.2955 (39.59), 278.2226 (23.13), 277.2195 (100), 242.0754 (4.85), 224.0676 (15.42)	Lysophosphatidylcholine	5.02
42	17.29	9-hydroperoxyl-11,12-octadecadienoic acid	$C_{18}H_{32}O_4$	$[M-H]^-$	311.223	312.230	-0.60	311.2219 (100), 293.2107 (23.12), 171.1030 (12.03)	Fatty acid	0.69
43	17.44	13-Hydroxy-9,11,15-octadecatrienoic acid	$C_{18}H_{30}O_3$	$[M-H]^-$	293.212	294.219	-1.95	293.2119 (23.9), 275.1999 (91.44), 231.2115 (11.75)	Fatty acid	0.21
44	17.48	PS(18:2)	$C_{24}H_{44}NO_9P$	$[M-H]^-$	520.269	521.276	0.56	520.2674 (3.15), 387.7386 (0.36), 152.9973 (100)	Glycerol ester	3.87
45	17.64	(9Z,12Z)-(7S,8S)-Dihydroxyoctadeca-9,12-dienoic acid	$C_{18}H_{32}O_4$	$[M-H]^-$	311.223	312.230	-1.00	311.2222 (100), 293.2123 (16.88), 171.1009 (9.85), 141.0924 (10), 125.0977 (19.52)	Fatty acid	0.90
46	17.80	LysoPE 18:2/ LysoPE (0:0/18:2)	$C_{23}H_{44}NO_7P$	$[M-H]^-$	476.280	477.287	3.56	476.2793 (4.21), 280.2378 (23.71), 279.2347 (100), 214.0458 (11.56), 196.0348 (1.29)	Lysophosphatidylethanolamine	17.38
47	17.92	LysoPC 18:2/ LysoPC (0:0/ 18:2)	$C_{26}H_{50}NO_7P$	$[M+HCOO]^-$	564.327	519.329	-7.47	564.3142 (0.59), 504.3101 (28.83), 280.2356 (25.51),	Lysophosphatidylcholine	19.81

								279.2316 (100), 242.0819 (5.2), 224.0722 (0.51)		
				$[M+H]^+$	520.341	519.329	-5.59	520.3514 (1.84), 184.0783 (100)	Lysophosphatidylcholine	10.52
48	17.96	LysoPE 15:0	$C_{20}H_{42}NO_7P$	$[M-H]^-$	438.261	439.269	-1.85	439.2555 (9.62), 242.215 (19.38), 241.2123 (100), 196.0345 (13.99)	Lysophosphatidylethanolamine	1.03
49	18.20	LysoPE 18:2(2n isomer)/ LysoPE (18:2/ 0:0)	$C_{23}H_{44}NO_7P$	$[M-H]^-$	476.280	477.287	3.88	476.2790 (9.3), 280.2378 (21.4), 279.2352 (100), 214.0459 (2.75), 196.0354 (12.35)	Lysophosphatidylethanolamine	6.67
50	18.21	12-Oxo-phytodienoic acid	$C_{18}H_{28}O_3$	$[M-H]^-$	291.196	292.203	-3.56	231.5276 (1.43), 205.0156 (2.73), 191.7374 (1.99), 127.0028 (100), 125.9953 (6.19)	Fatty acid	0.08
51	18.30	LysoPC 18:2(2n isomer)/ LysoPC (18:2/ 0:0)	$C_{26}H_{50}NO_7P$	$[M+HCOO]^-$	564.333	519.334	3.49	564.3356 (0.52), 504.3102 (44.13), 280.238 (22.91), 279.2350 (100), 242.0805 (2.65), 224.0711 (12.72)	Lysophosphatidylcholine	4.69
				$[M+H]^+$	520.341	519.334	3.06	520.3519 (18.85), 184.0784 (100)	Lysophosphatidylcholine	4.86
52	18.74	LysoPE 16:0	$C_{21}H_{44}NO_7P$	$[M-H]^-$	452.278	453.286	-0.10	452.2705 (7.53), 255.2347 (100)	Lysophosphatidylethanolamine	1.00
53	18.81	9-hydroxyoctadecadienoic acid (9-HODE)	$C_{18}H_{32}O_3$	$[M-H]^-$	295.228	296.235	-1.32	295.2237 (58.96), 277.2141 (100), 171.1004 (69.98)	Fatty acid	0.01
54	19.30	13-hydroxyoctadecadienoic acid	$C_{18}H_{32}O_3$	$[M-H]^-$	295.227	296.234	-4.17	295.2237 (59.3), 277.2143 (100), 195.1371 (15.51)	Fatty acid	0.43
55	19.32	LysoPE 16:0(2n isomer)	$C_{21}H_{44}NO_7P$	$[M-H]^-$	452.280	453.287	3.47	452.2802 (9.41), 255.2347 (100)	Lysophosphatidylethanolamine	2.21
56	19.68	2-Linoleoylglycerol-1-O-glucoside or isomer	$C_{27}H_{48}O_9$	$[M+HCOO]^-$	561.329	516.331	1.47	516.3123 (0.66), 280.2386 (23.35), 279.2345 (100), 253.0944 (16.66)	Glycerol ester	6.51
57	19.89	13-Hydroxy-6,9,11-octadecatrienoic acid	$C_{18}H_{30}O_3$	$[M-H]^-$	293.212	294.219	-0.99	293.2117 (100)	Fatty acid	0.38
58	20.46	(9Z,11E)-13-Oxooctadeca-9,11-dienoic acid	$C_{18}H_{30}O_3$	$[M-H]^-$	293.212	294.219	-0.55	293.2139 (100)	Fatty acid	0.61
59	20.71	Tokoronin	$C_{32}H_{52}O_9$	$[M+Na]^+$	603.347	580.357	-6.25	603.3432 (100), 441.2925 (39.49)	Terpenoid	0.17
60	24.86	(9Z,12Z)-(7S,8S)-Dihydroxyoctadeca-9,12-dienoic acid	$C_{18}H_{32}O_3$	$[M-H]^-$	295.228	296.235	-1.44	295.2278 (100) 249.2221 (54.82)	Fatty acid	0.03

61	26.34	α-Linolenic acid	$C_{18}H_{30}O_2$	$[M-H]^-$	277.217	278.224	-1.16	277.2126 (100)	Fatty acid	0.58
62	26.37	2-Hydroxyhexadecanoic acid	$C_{16}H_{32}O_3$	$[M-H]^-$	271.229	272.236	2.79	271.2277 (43.83), 225.2225 (100)	Fatty acid	0.05
63	26.70	Ricinoleic acid	$C_{18}H_{34}O_3$	$[M-H]^-$	297.243	298.250	-2.47	297.2428 (54.76), 251.2390 (100)	Fatty acid	0.06
64	27.40	3-Hydroxyoctadecanoic acid	$C_{18}H_{36}O_3$	$[M-H]^-$	299.258	300.266	-2.87	299.2573 (59.16), 253.2544 (100)	Fatty acid	0.79
65	27.40	Palmitaldehyde	$C_{16}H_{32}O$	$[M+CH_3COO]^-$	299.258	240.245	-3.59	99.9226 (100), 68.9944 (35.98), 55.0181 (29.29)	Fatty acid	0.17
66	27.60	Kadsurenone or isomer	$C_{21}H_{24}O_5$	$[M-H]^-$	355.157	356.163	2.28	355.1533 (24.17), 100.9344 (33.38)	Lignans	1.00

Table S3 Summary for PCA and PLS-DA models

Component	R^2X (cum)	Q^2 (cum)	R^2Y
PCA			
1	0.693	0.574	-
2	0.766	0.658	-
3	0.877	0.724	-
4	0.964	0.709	-
PLS-DA			
1	0.490	0.694	0.594
2	0.756	0.799	0.775
3	0.842	0.892	0.906
4	0.913	0.899	0.962
